# Allergic reactions to insulin: a case report

**DOI:** 10.1186/2045-7022-4-S3-P4

**Published:** 2014-07-18

**Authors:** Rita Aguiar, Natalia Fernandes, Ana Mendes, Manuel Pereira-Barbosa

**Affiliations:** 1 Hospital de Santa Maria-Centro Hospitalar Lisboa Norte, Immunoallergology Department, Portugal

## 

We present the case of a woman, 47 years old, with type 1 diabetes mellitus. She was regularly treated with detemir insulin (LevemirTM) tid, lispro protamine (Insulin Humalog Mix 25TM) according with glucose during the day. She noticed the appearance of non-confluent urticariform lesions on the thorax and upper limbs, totalling 20, with a pruriginous papule at the site of subcutaneous insulin Levemir injection after 4-6 hours. The lesions spontaneously regressed after 24 hours of the administration-biphasic reaction. No other systemic manifestations or triggers were verified. She had partial improvement with a non-sedating H1 antihistamine. Laboratory evaluation was negative for other autoimmune diseases. Total IgE <0.1 UI/mL, serologies for Cytomegalovirus, Epstein-Barr virus, coxsackievirus, echovirus, HIV 1/2, hepatitis B/C virus were also negative. High serum glycated hemoglobin A1c levels (7.8%) were found and apparently correlated with the cutaneous reaction. Skin tests were carried out with Levemir insulin (100U/ ml, 3,5 mg/ml): negative prick test, intradermal skin test 1/100, 1/10 and pure, showed a papule equal to the initial one but with an extensive surrounding erythema. Humalog insulin (100U/ml, 3,5mg/ml): negative prick test, intradermal skin test 1/100, 1/10 and pure, with a papule equal to the initial one without surrounding erythema. Pure protamine sulfate 10mg/ml with negative results. Specific IgE: IgE human insulin<0.10 KU/L, bovine insulin IgE<0.10KU/L, porcine insulin IgE<0.10 KU/L, human insulin specific IgG <2. Given the results, it was decided to perform skin tests with an alternative Insulin:Lantus Insulin-glargina (100UI/ml-3,64mg) with negative results. Insulin Levemir was suspended and the patient began therapy with Lantus insulin, with no reactions. Glycemic control was achieved after three months.The patient stopped H1 antihistamine with no recurrence of urticaria. Allergy to insulin analogues is rare and requires early diagnosis, leading to a major therapeutic challenge. Despite the of suspicion of insulin hypersensitivity, it should be mantained until alergic investigation is complete and a safe alternative can be found (if not, insulin desensitization can be a therapeutic option).

